# Supplementary material for: Comparative efficacy of chemical and botanical pediculicides in Thailand and 4% dimeticone against head louse, Pediculus humanus capitis
Source: PLoS One. 2023 Jun 23;18(6):e0287616. doi: 10.1371/journal.pone.0287616 (PMC10289457; doi:10.1371/journal.pone.0287616)
Supplement: S1 File — (DOCX) [file pone.0287616.s001.docx]

**Table 1.** Percentage mortality of head lice recorded at 30 min and 8 hr after the end of a 10 or 15 min exposure to each solution based on the topical test.

| **Tests** | **Observation time**  **Mean no. death/ total ±SE (Mortality (%)**) | |
| --- | --- | --- |
|  | **30 min** | **480 min (8 hr)** |
| A: 1% permethrin (reference control) | 0/10± 0.00 (0) | 0/10± 0.00 (0) |
| B: 0.6% carbaryl | 3.3/10± 0.15 (33.3)** | 5.0/10± 1.00 (50.0) ** |
| C: Mixed plant extracts | 0.6/10± 0.58 (6.6)*^NS^* | 0.6/10± 0.58 (6.6) *^NS^* |
| D: 0.15% *Stemona* root crude extract | 0/10± 0.00 (0)*^NS^* | 0/10± 0.00 (0) *^NS^* |
| E: 4% dimeticone (Hedrin (10 min) | 9.67/10± 0.58 (96.7)** | 9.67/10± 0.58 (96.7)** |
| 4% dimeticone (Hedrin) (15 min) | 10.0/10± 0.00 (100)** | 10.0/10± 0.00 (100) ** |
| Distilled water (negative control) | 0/10± 0.00 (0)*^NS^* | 0/10± 0.00 (0) *^NS^* |

*The experiments were conducted in triplicate (n= 10 each group).*

*NS= not significant; * = p <0.01; ** = p <0.001 based on Kruskal–Wallis and Dunn's test (compared mean number of lice with reference control group: 1% permethrin at 8 hr)*

**Table** **2.** Percentage mortality of head lice recorded 30 min and 8 hr after the end of a 30 min exposure to each solution based on the topical test.

| **Tests** | **Observation time**  **Mean no. death/ total ±SE (Mortality (%)**) | |
| --- | --- | --- |
|  | **30 min** | **480 min (8 hr)** |
| A: 1% permethrin (positive control) | 4.3/10± 0.58 (43.3) | 4.3/10± 0.58 (43.3) |
| B: 0.6% carbaryl | 6.0/10± 1.00 (60.0)** | 6.0/10± 1.00 (60.0) ** |
| C: Mixed plant extracts | 1.7/10± 1.15 (17.0)** | 1.7/10± 1.15 (17.0) ** |
| D: 0.15% *Stemona* root crude extract | 2.0/10± 0.00 (20.0)* | 2.0/10± 0.00 (20.0) * |
| E: 4% dimeticone (Hedrin) | 10.0/10± 0.00 (100)** | 10.0/10± 0.00 (100) ** |
| Distilled water (negative control) | 0/10± 0.00 (0)** | 0/10± 0.00 (0) ** |

*The experiments were conducted in triplicate (n= 10 each group).*

*NS= not significant; * = p <0.01; ** = p <0.001 based on Kruskal–Wallis and Dunn's test (compared mean number of lice with reference control group: 1% permethrin at 8 hr)*
