# Supplementary material for: Comparative efficacy of chemical and botanical pediculicides in Thailand and 4% dimeticone against head louse, Pediculus humanus capitis
Source: PLoS One. 2023 Jun 23;18(6):e0287616. doi: 10.1371/journal.pone.0287616 (PMC10289457; doi:10.1371/journal.pone.0287616)
Supplement: Attachment — Submitted filename: Response to editor and reviewers PONE-D-23-11571-R1.docx [file pone.0287616.s003.docx]

Response to reviewers

PONE-D-23-11571

Comparative efficacy of chemical and botanical pediculicides in Thailand and 4% dimeticone against head louse, *Pediculus humanus capitis*

We are grateful for the opportunity provided by the editor and reviewers to revise the manuscript. We sincerely appreciate the reviewers' insightful criticism and valuable recommendations, which have greatly contributed to improving the quality of the manuscript. All of the reviewers' suggestions and comments have been thoroughly addressed.

Reviewer #1:

Lines 32-33: It might be better to give a range of percentage mortality

**Response:** We have provided a range of percentage mortality as “…..whether treatment was for 10 min (resulting in 0 to 50.0% mortality) or 30 min (resulting in 17.0 to 60.0% mortality)……..”

Lines 35-36: Are you saying that none of the 26 children treated with 1% permethrin was cured after two treatments?

**Response:** Yes, we have modified the sentence as “none of the 26 children treated with 1% permethrin shampoo achieved a cure after two applications.”

Lines 50-51: It does not appear in this publication that body louse feeds once or twice a day!

**Response:** We acknowledge this comment and have modified to “body louse feeds about five times a day”.

Lines 52-53: Does it mean that body lice were never found in Thailand?

**Response:** Yes, there aren’t any previous reports of body lice being found in Thailand. We have modified the sentence as “ The human louse found in Thailand is the head louse: *Pediculus humanus capitis* [6, 7], while body louse has never been reported.”

Lines 69-70: in the reference number 20 it is rather said that…. : In treating head louse infestation, evidence suggests occlusive agents may be superior to or equally efficacious as neurotoxic pediculicides.

**Response:** We have modified accordingly and also included additional references.

“To combat resistant lice, it is essential to explore treatment options utilizing medications from different classes [20]. One potential alternative is the use of physically acting agents, with dimeticone being a prominent option, particularly in regions with a high resistance rate [21]. It has been suggested that dimeticone is superior to or equally effective as neurotoxic pediculicides [22].”

Line 114: Pediculicides used might be better than “Chemical preparation”

**Response:** We have changed “Chemical preparation” to “Pediculicides used” accordingly.

Line 121: Was only one kind of louse comb used and if yes if you could give any details about the comb

**Response:** We have added more details of the louse comb as “….using a fine-tooth comb (with teeth spaced 0.3 mm apart and 10.7 mm in length)….”

Lines 254-: It is suggested that the conclusion should be stronger than written here and should include that the most sold and authorized by the health authorities pediculicides are not effective, which would also explain the high rate of infestation of children in the country and it would be advisable to the health authorities to introduce dimethicone-based products such as Hedrin ASAP to the country.

**Response:** We have revised it according to the suggestions provided by the reviewer in the conclusion section.

Reviewer #2: The manuscript presents the outcomes of an important study in Thailand, where head lice infestation (HLI) is a significant problem among primary school children, as in many parts of the world. The study is mostly well-designed and well-written and its outcomes are significant as they include the first assessment results of dimeticone in Thailand, presents ex vivo results of local products used in HLI, together with efficacy of permethrin 1% among school children. The content of the study is very rich and there is a hard work in the background of the study. However, an important mistake seems to be effective in the results of HLI-positive school children investigation with 1% permethrin. The study universe had 109 primary school girls only, not any boys. In our experience, despite their short hair compared to girls, boys can also harbor head lice and transmit them to their classmates. As the boys were not investigated nor treated with 1% permethrin on Day 1 and 7, the HLI-positive girls identified on days 7 and 14 may be due to reinfestation from their classmate boys, not because of permethrin resistance. Since permethrin resistance have been reported from many regions in the world, it is not surprising to identify such a resistance also in Thailand, but the design of the study does not exclude the risk of reinfestation from classmates. This potential risk should be mentioned within the drawbacks of the study.

**Response:** We acknowledge your insightful comments on our manuscript. We also appreciate your attention to detail and the valuable points you have raised regarding the potential risk of reinfestation from classmates, particularly boys, in our study on HLI-positive school children treated with 1% permethrin.

We understand your concern that boys, despite having shorter hair compared to girls, can also harbor head lice and transmit them to their classmates. However, it is important to note that previous studies conducted in Thailand have reported a very low or no infestation rate among boys, suggesting that their contribution to the reinfestation dynamics in our study population may be minimal. Nevertheless, we have included the importance of considering potential reinfestation sources, such as classmates in our limitation section to provide a comprehensive analysis of the findings and emphasize the importance of considering this aspect in future investigations as suggestion in the Discussion.
